# T Cell Therapy Targeted on HLA-A02 Restricted HIV Antigen Epitopes: An Open Label Cellular Therapy Trial Using CD8+ T Cell

**DOI:** 10.3389/fimmu.2019.00437

**Published:** 2019-03-18

**Authors:** Sai Liu, Jianping Sun, Zhen Li, Ling Qin, Guihai Liu, Kang Li, Hao Wu, Tao Dong, Yonghong Zhang

**Affiliations:** ^1^Biomarkers of Infection Related Diseases Beijing Key Laboratory, Beijing You'An Hospital, Capital Medical University, Beijing, China; ^2^Center of Infectious Disease, Beijing You'An Hospital, Capital Medical University, Beijing, China; ^3^Medical Research Council Human Immunology Unit, Weatherall Institute of Molecular Medicine, John Radcliffe Hospital, Oxford University, Oxford, United Kingdom

**Keywords:** T cells, cellular immunity, antigen epitopes, cellular therapies, HIV, HLA-A02

## Abstract

**Objective:** To test the safety and efficacy of a T cell therapy *de novo* targeting HLA-A02 restricted HIV antigen epitopes.

**Design:** This was a prospective open label clinical trial, which enrolled 28 HIV+ participants and 24 of them finished the trial. The study was publicly registered at Chinese Clinical Trial Registry, www.chictr.org.cn(ChiCTR-ICR-15005775).

**Method:** Autologous peripheral blood mononuclear cells were co-cultured with HLA-A02 restricted HIV antigen epitopes peptides to produce cell product for this therapy. The trial was divided into five time-points with the same interval period for infusion of the cell products or monitoring parameters. Symptoms, vital signs, and blood samples were collected to analyze the safety and efficacy of this therapy.

**Results:** Two cases of adverse effects happened during this trial in test group, which recovered without medical intervention. There was no severe adverse effect that occurred. Both symptoms and laboratory tests have no statistical significant difference between test and control group. Flowcytometry analysis showed the expression of the PD-1 and CD95 molecule on the cell surface were downregulated post-treatment in the test group.

**Conclusions:** This autologous HIV-antigen specific effector CD8+ T cellular therapy was safe. It might have an impact on immune suppression that can provide useful reference to future cell therapy trials.

## Introduction

The HIV/AIDS epidemic has been one of the most important global public health issues for more than 40 years. The current treatment for this infection, the combined antiretroviral therapy (cART), is able to suppress the HIV viremia to an undetectable level long-term, thus tremendously prolonging the life expectance of the patients. However, this therapy requires a lifelong adhesion to it, and puts a huge burden on the patients and the healthcare authorities.

The reason why cART cannot eradicate HIV infection is because firstly, it cannot infiltrate certain body compartments, such as the lymph node and the central nerve system (1). Secondly, latent HIV pre-virions remain combined to the genome of the infected cells but not producing new viral particles (2). Soon after treatment cessation, the HIV viremia will relapse.

The investigation for eradicating this virus has never stopped. With the discovery of the long-time-no-progressor (LTNP) and elite-controller of HIV, it is known now that the natural immune system could have the ability to control the infection on its own. Previous studies have shown that CD8+ cytotoxic lymphocytes (CTLs) played a central part in controlling HIV viremia by the immune system, especially in the rapid decline of viral load in the late stage of acute HIV infection (3). But for CTLs to perform this function, it starts with the recognition of HLA restricted viral epitopes. During the early stage investigation of LTNP and elite controller, it was found that some HLA subtypes are protective against the progression of this infection (4, 5). The mechanism behind this phenomenon is not very clear, but one theory is that some of these restricted epitopes are crucial parts of viral protein, and the mutation of this epitope would compromise the replicational function of the virus. Later on, it was highlighted that the exhaustion of T cells in proliferation and/or immune activity contribute greatly to the loss of viremia control (6, 7). Along with the traditional mechanism of cell apoptosis markers, if the immune check-point molecule is unfavorably regulated, cell function in proliferation, survival, or secretion can be damaged. These findings provoked the interest to investigate the potential of cellular/immune therapy to cure HIV infection.

Under these circumstances, and based on previous studies we performed, our team designed and conducted this cellular therapy trial which uses HLA-A02 restricted HIV-antigen specific CTLs as effectors cells, to investigate the safety and any potential efficacy of this cellular therapy.

## Methods and materials

### Participants

The trial was performed in Beijing You'An Hospital. Participants were from the You'an cohort of treatment at acute phase of HIV infection. All the participants in this cohort were able to identify their event of transmission and have commenced combined anti-retroviral therapy (cART) within 6 months of HIV infection. Participants had to meet the following criteria to be recruited: (1) age between 18 and 60 years; (2) HIV antibody positive by diagnostic guideline; (3) possessing HLA-A02 alleles. (4) HIV Viral load <50 copies/ml; (5) do not have severe HIV directly or indirectly related complications; (6) capable of understanding the objection, method, possible adverse events, and their medical interventions, accept the duty and right of participating in this trial.

After enrollment, the participants were assigned into test group and control group. Randomization of assignment was performed in a Microsoft Excel software by generating a random number.

Design and conduction of the trial was according to the Declaration of Helsinki. Ethic issues were reviewed and approved by the Ethic Review Board of Beijing You'An Hospital and Sun-Yat-Sen University. Written informed consent was obtained from every participant, as appropriate, before starting any study-related procedure. The consent paperwork included the explanation of the trial, potential side effects, and their medical interventions, right and duty of the participants, and consent for publishing their anonymized data. The study was publicly registered at Chinese Clinical Trial Registry, www.chictr.org.cn (ChiCTR-ICR-15005775).

### Overall Procedure of the Trial

The trial has been divided into 5 time-points with an equal interval of 2 weeks, namely baseline, first infusion, second infusion, first follow-up, second follow-up.

At the beginning of every time-point, vital signs (heart rates, blood pressure, weight, height) of all the participants were recorded and blood samples were acquired for laboratory tests and blood cell/serum separation then stored in liquid nitrogen. In addition, participants also went through a chest X-Ray test and an abdominal ultrasonography for accompanying major diseases at the baseline.

For test group, part of their blood samples was used to manufacture the cell product at baseline and first infusion time-point, and then infused back into the patients at first and second infusion, respectively. Each participant would be observed for 4 h after infusion for the purpose of monitoring vital signs and symptoms. The control group would not receive cell product, but the same vital signs, symptoms and laboratory works as the test group would be recorded/performed.

**Figure 1:**
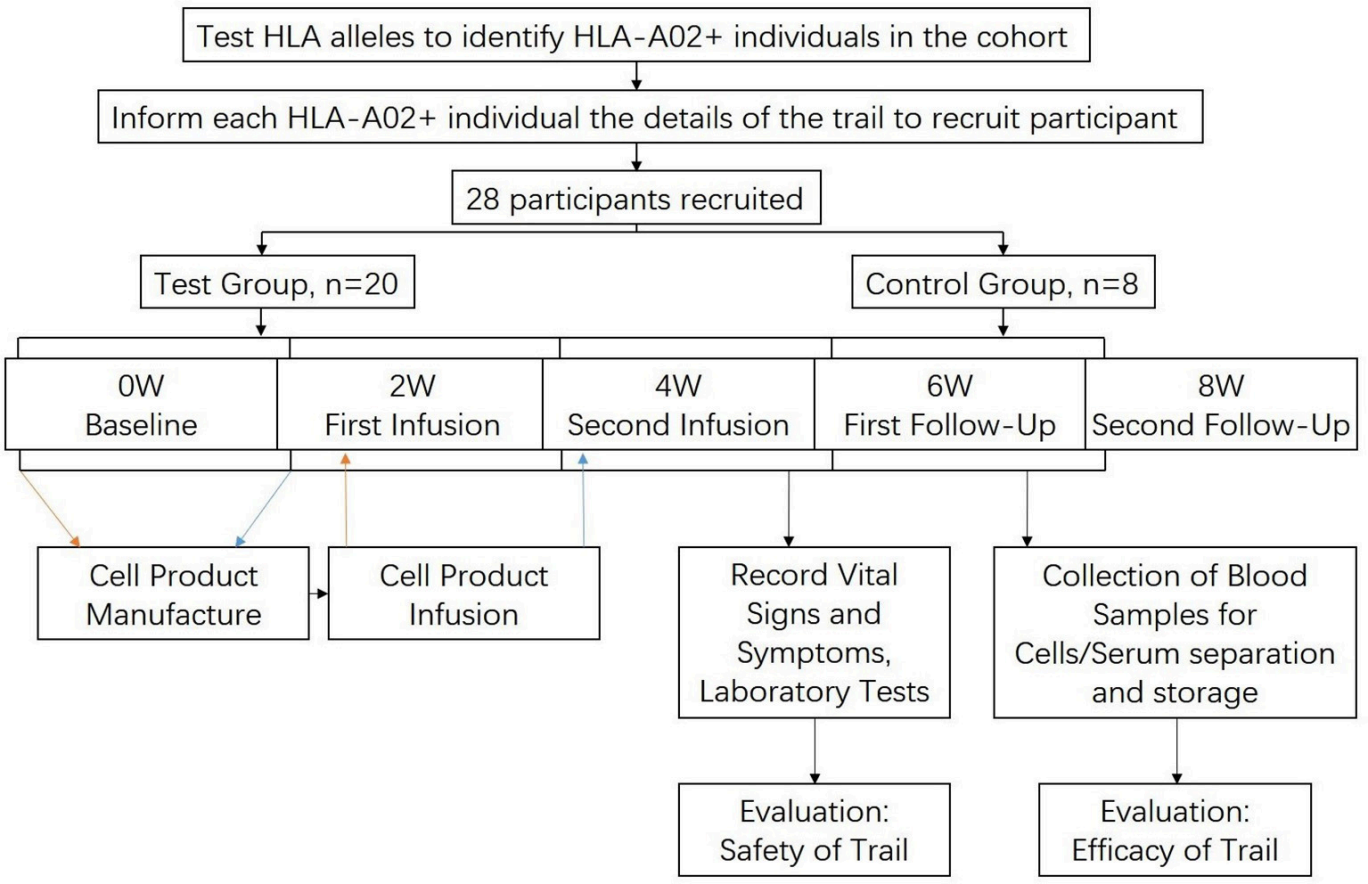
Flow chart showing the procedure of the trial.

When the trial was finished, some of the stored PBMC of the test groups were used to go through flowcytometry to measure their changes of surface markers, to see the efficacy of the therapy. The procedure of the trial is visualized in Figure 1.

### Cell Products: Character and Procedure

For each product, the autologous PBMCs were co-cultivated with HLA-A02 restricted HIV antigen epitopes acquired from the database of Los Alamos laboratory, the sequence of every epitope is listed in Table 1. All epitope peptides synthetized by Weatherall institute of molecular medicine, University of Oxford, UK. The cell cultures were performed in a non-serum cell cultivation medium environment (serum free medium CCM, GIBCO®, ThermoFisher Co. Ltd., USA) with IL-2 (500 U/ml) as cytokine support, in order to minimize potential contamination and allergic reaction.

**Table 1:** Name and sequence of HLA-A02 restricted epitopes used in the cell products manufacture.

**Epitope name**	**Epitope sequence**
HIV-Pol-AM9	ALVEICTEM
HIV-Pol-YI9	YTAFTIPSI
HIV-Pol-VL9	VIYQYMDDL
HIV-Pol-IV9	ILKEPVHGV
HIV-Gag-SL9	SLFNTVATL
HIV-Gag-IV9	ILAEAMSQV
HIV-Gag-FK10	FLGKIWPSYK
HIV-Nef-PL10	PLTFGWCYKL
HIV-Nef-VL10	VLEWRFDSRL

The manufacture of the products was conducted in a good manufacture product (GMP) certificated laboratory. The duration of the culture was around 14 days. At the end of the culture, supernatant of every culture was used to test HIV-P24 antigen level, bacteria contamination and pyrogen level. The cultured cells were washed and centrifuged then resuspended with 50 ml nature saline supplied with 10% albumin for infusion. The detailed method and cell product profile have already been published (8).

### Safety Monitoring

#### Definition of Adverse Events (AE) and Severe Adverse Events (SAE)

In this trial, AE/SAE were defined in accordance to the Common Toxicity Criteria (Ver. 4.0) published by World Health Organization and National Cancer Institute. Specific conditions and symptoms may have values or a descriptive comment for each level, graded as Mild, Moderate, Severe, Life threatening and Death, or grade 1–5, respectively. In this trial, we regarded grade 1 and 2 as adverse events and generally would not need medical intervention, grade 3–5 as severe adverse events and would be medically intervened.

#### Symptoms and Vital Signs

At the beginning of all five time-points, the participants were asked about every symptom they had during two time-points, especially for newly emerged symptoms. Vital signs were recorded, including heart rate, blood pressure, weight and height.

For the test group, participants would be monitored for 4 h after the initiation of infusion. Blood pressure would be recorded at 0.5 and 4 h after the infusion. Symptoms, especially allergic reactions such as pruritus, rush and edema, were observed.

#### Laboratory Test

At the beginning of every time-point, blood sample would be collected from all the participants for laboratory tests. These tests included count of blood cells, biochemistry panel, HIV viral load, and count of immune cells. Crucial results in these tests were recorded for analysis which is shown in the result section. All these tests were performed by the Clinical Laboratory Department of Beijing You'An Hospital.

### Efficacy Test

#### Samples

Apart from the clinical laboratory tests, 60 ml of blood was acquired from each participant and used for PBMCs isolation which were then stored in liquid nitrogen. At the end of the trial, part of the PBMCs were used to test the change of cell surface marker to identify the efficacy of the therapy. The cells of the baseline or first infusion time-point were regarded as the pre-treatment condition, as at these time-points the participants were not exposed to the cell product. Blood sample of the first follow-up time-point were regarded as the acute post-treatment condition, as it was the first sample after 2 times of cell product infusion. Changes of cell surface markers were identified by flowcytometry (Fortessa flowcytometer, BD, USA) in both groups, percentages of the expression of each marker tested were then analyzed.

#### Flowcytometry: Three Panels

Three panels of flowcytometry were performed to identify different functions of the cells. These panels were named immune check point panel, differentiation panel and activation/apoptosis panel.

All panels contained antibody/dye of CD3, CD4, CD8, and aqua live or dead. All the detailed information of the antibodies used in this trial is listed in Supplementary Table 1.

Before staining, antibody cocktail were made by mixing all antibody for each panel and diluted to 100 μl volume with PBS solution per single dose. Every single dose of antibody cocktail contained 5 μl (1 test) of each antibody. The incubation period of each panel was 1 h to ensure simultaneous end of incubation. Florescence minus one (FMO) was used to determine the cut-off gating strategy, which is shown in Supplementary Figure 1. Gating strategy for subset of CD8+ T cells analysis is shown in Supplementary Figure 2.

### Statistical Analysis

In this trial, data were measured in quantified values. For baseline evaluations and efficacy test, analyses were performed using the *t*-test (normal distribution data) or the non-parametric Wilcoxon matched pair analysis (skewed distribution data). For symptoms, the number of events in both groups were compared using Chi Square test. For laboratory test, comparisons were between the five time-points of both groups using two-way ANOVA test, the purpose of the comparison was to analyze the difference of the trend of laboratory parameters between the two groups. Data is presented either in mean ± standard deviation (normal distribution data), or median, 25% quartile and 75% quartile (skewed distribution data).

## Result

### Profile of Participants

There were 28 individuals who met the criteria and were willing to participate in this trial. Twenty of them were assigned to the test group and eight to control group. Until the end of the trial, three in test group and one in control group were lost to follow-up due to poor adhesion.

All the participants were male, have already initiated cART and were with undetectable HIV viral load. Clinical profile of the participants was recorded, and statistical analysis was performed to test the difference between the two groups of baseline data. For the laboratory parameters used in safety test, there was no statistic significant difference (Table 2).

**Table 2:** Clinical profile of the participants and their baseline data of the two groups.

	**Test group**	**Control group**	***p-*value**
Total Pt.	17	7	NA
Gender (male/female)	17/0	7/0	NA
Age (mean ± SD)	37.47 ± 10.24	36.88 ± 7.415	NS
Commenced cART	17	7	NA
Duration of cART (month, mean ± SD)	19.24 ± 16.79	22.88 ± 19.92	NS
Viral load (<LDL^*^/Detectable)	17/0	7/0	NA
CD4+ T cell (ml/μL)	529.9 ± 228.8	497.8 ± 202.1	NS
CD8+ T cell (ml/μL)	821.2 ± 438.7	641.6 ± 236.6	NS
WBC (count/L)	4.97 [4.79, 5.72]	5.35 [5.19, 5.54]	NS
HGB (g/L)	151.4 ± 10.91	152.6 ± 11.59	NS
LYM(count/L)	1.80 ± 0.65	1.82 ± 0.58	NS
ALT (IU/L)	43.01 ± 33.2	54.49 ± 38.07	NS
AST (IU/L)	24.2 [21.23, 35.53]	26.4 [22.2, 44.5]	NS
Tbil (μmoI/L)	11.65 [9.25, 15.18]	10.5 [8.3, 10.9]	NS
Cr (μmoI/L)	67.25 [62.55, 72.9]	64.7 [55.2, 72.6]	NS
GLU (mmol/L)	4.482 ± 0.6561	4.203 ± 0.3287	NS
CK (mmol/L)	102.4 [74.05]	83.1 [71.3, 111]	NS

* *LDL, Lower detectable level, 50 copies/ml*.

*NA, Not Applicable*.

*NS, Non-significant*.

### Cell Product Profiles

On the day of product packaging, all the cell products went through test of total cell counting, endotoxin and HIV-p24 antigen level to analyze overall cell profile and ensure the safety of the products. After these, cells in the culture would be washed with nature saline and re-suspended to 50 ml with natural saline-albumin solution and infused back to the patients.

Cells in the products gained an average of 13.67(ranging 3–46.7)fold expansion post-culture. In addition, intracellular cytokine stainings (ICS) for interferon-γ were performed on pre- and post-cultivation to test the antigen specificity of the cell expanded. The result showed the average count of HIV antigen specific CD3+CD8+IFN-γ+ cells was 13.75(ranging 0–109.8) × 10^5^. Detailed information has been provided here in Supplementary Table 2.

### Safety: Adverse Events/Severe Adverse Events (AE/SAE) for Vital Signs, Symptoms, and Laboratory Tests

In the test group, there were 2 AE incidence, no SAE happened. The AEs were: (1) 1 case of lower limb pruritus, without any rush. It was not medically intervened, ceased within 8 h. (2) 1 case with 30 mmHg increase of systolic blood pressure which had no related symptoms. It was not medically intervened and the systolic blood pressure dropped to baseline level at the next time-point. In control group there were no AE/SAE happened. Data were analyzed by Chi square test, no statistical significance was found (*p* = 0.3579, Table 3A).

**Table 3a:** Safety: symptomatic adverse effect comparison.

	**AE/SAE**	**No AE/SAE**	***p-*value**
Test group (case)	2	15	
Control group(case)	0	7	NS

For the effects on immune cell subtypes, there was no severe acute depletion of CD3+CD4+ T cells in both groups, both CD3+CD4+ T cell and CD3+CD8+ T cells were steady through the trial (*p* = 0.9648, 0.4028, respectively). No relapse of HIV viremia observed during the trial.

For the effects on blood cells, quantitative value of total white cell count (WBC), Hemoglobin (HGB), and lymphocyte count (LYM) were compared between the two groups. The result showed no statistic difference between the two groups through the 5 time-points (WBC *p* value = 0.3836, HGB *p* value = 0.6594, LYM *p* value = 0.9565), thus there was no effect on the targeted blood cell compartments.

**Figure 2:**
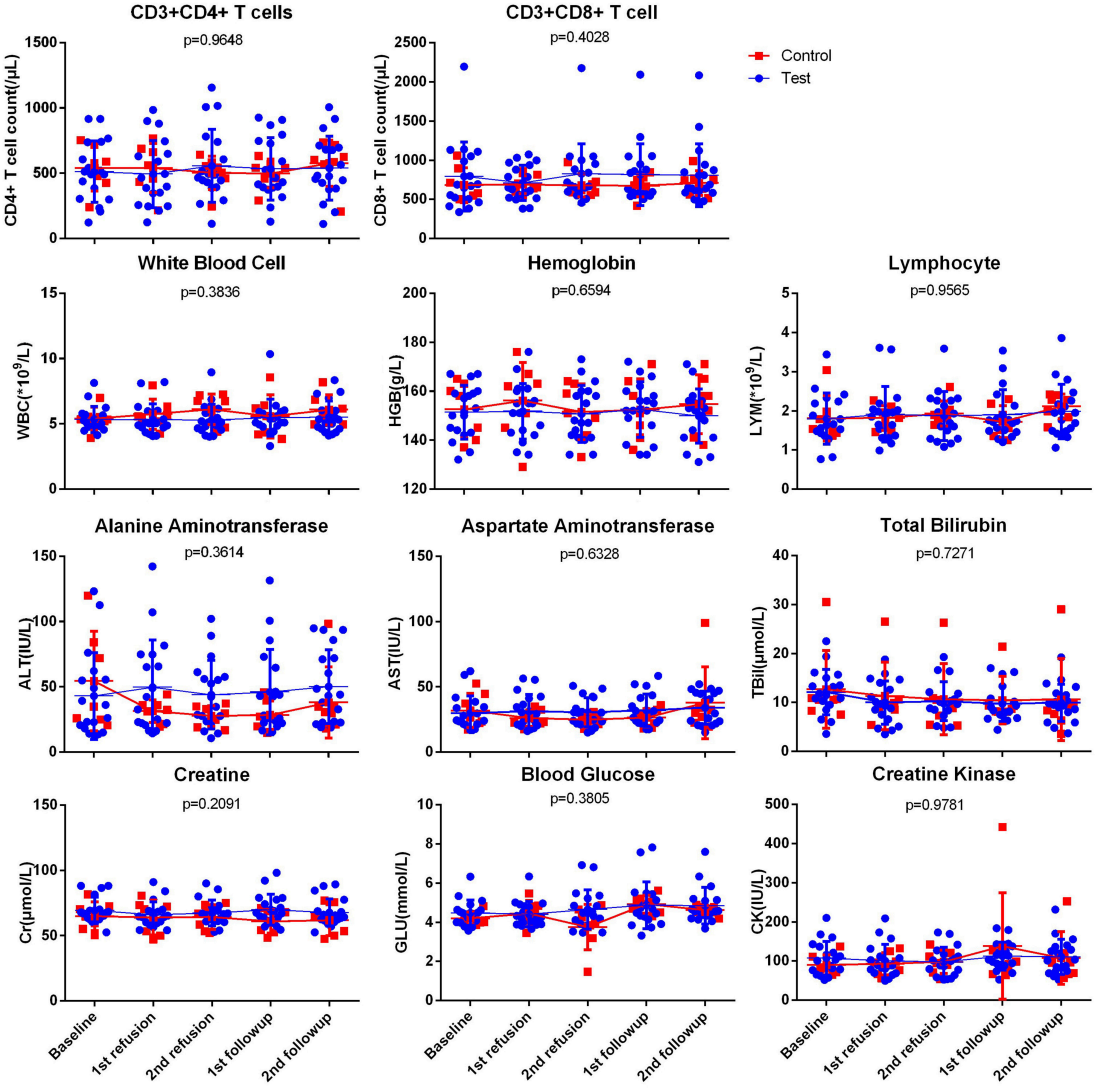
The changes of all the parameters of laboratory evaluations of safety. Each dot represents an individual data, the horizontal line on each set of data represents the standard deviation of the values. WBC, White blood cell; HGB, Hemoglobin; LYM, Lymphocyte; ALT, Alanine Aminotransferase, AST, Aspartate Aminotransferase; TBil, Total Bilirubin; Cr, Creatine; GLU, Blood Glucose; CK, Creatine Kinase.

Like the effects upon blood cells, data of biochemistry panel showed no difference of liver and renal function test. Comparison of alanine aminotransferase (ALT), aspartate aminotransferase (AST), total bilirubin (TBil), and creatinine (Cr) through the trial showed no significance between two groups (*p* value = 0.3614, 0.5384, 0.7271, 0.2735, respectively). Level of Creatine Kinase (CK) showed no significance as well (*p* value = 0.9781). Notably, Although the trend of blood glucose (GLU) level showed no difference between the two groups (*p* value = 0.3805), one case of hyperglycemia occurred during the trial in test group. The lifestyle of the participant was irrelevant to this situation, but in accordance with the steady and even trend of blood glucose in test group, this occurrence is not considered relevant to the therapy. Overall, the result indicated that this therapy had no notable effect on liver and renal function, nor did it show induction of hyperglycemia and CK elevation related situation, such as muscle damage (Table 3B and Figure 2).

**Table 3b:** Safety: the changes of all the parameters of laboratory evaluations.

**Parameter**	**Group**	**Baseline**	**1st refusion**	**2nd refusion**	**1st follow-up**	**2nd follow-up**	***p-*value**
CD4+ T cell (count /μL)	Test	511.8 ± 234.8	493.9 ± 257.6	557.2 ± 279.3	534.7 ± 239.4	539.2 ± 243.9	
	Control	794.4 ± 440.6	712.1 ± 225.5	827.4 ± 383.3	816.7 ± 392.7	808.4 ± 401	NS
CD8+ T cell (count /μL)	Test	539.6 ± 177	540 ± 187.8	503.9 ± 128	498.6 ± 110	576.9 ± 175.7	
	Control	685 ± 218.5	689.7 ± 112.9	678.4 ± 144.7	675.4 ± 141	710 ± 157.6	NS
WBC (count/L)	Test	5.34 ± 0.99	5.33 ± 1.20	5.30 ± 1.21	5.48 ± 1.42	5.52 ± 1.25	
	Control	5.40 ± 0.90	5.75 ± 1.15	6.12 ± 1.17	5.58 ± 1.66	6.08 ± 1.15	NS
HGB(g/L)	Test	151.4 ± 10.91	151.8 ± 11.37	150.8 ± 11.6	152.6 ± 11.38	149.9 ± 11.04	
	Control	152.6 ± 11.59	156.1 ± 15.71	151.6 ± 11.43	152.4 ± 12.61	154.7 ± 12.02	NS
LYM (count/L)	Test	1.80 ± 0.65	1.92 ± 0.71	1.87 ± 0.63	1.90 ± 0.64	1.98 ± 0.69	
	Control	1.82 ± 0.58	1.82 ± 0.31	1.94 ± 0.32	1.72 ± 0.41	2.11 ± 0.32	NS
ALT (IU/L)	Test	43.01 ± 33.2	49.67 ± 36.1	43.99 ± 26.27	46.05 ± 32.74	50.23 ± 28.32	
	Control	54.49 ± 38.07	31.41 ± 8.618	27.8 ± 7.935	28.41 ± 10.83	38.1 ± 27.27	NS
AST (IU/L)	Test	29.9 ± 13.3	31.29 ± 12.87	30.57 ± 11.14	32.08 ± 12.29	34.07 ± 12.6	
	Control	31.96 ± 13.04	26 ± 6.28	24.93 ± 4.39	26.39 ± 6.94	34.39 ± 30.47	NS
Tbil (μmoI/L)	Test	12.12 ± 4.67	10.01 ± 4.41	10.26 ± 3.97	9.73 ± 3.62	9.97 ± 3.80	
	Control	12.7 ± 7.96	11.33 ± 6.90	10.66 ± 7.25	10.46 ± 4.86	10.63 ± 8.38	NS
Cr (μmoI/L)	Test	69.34 ± 10.14	66.02 ± 9.882	67.37 ± 9.866	69.91 ± 11.86	67.67 ± 10.44	
	Control	64.93 ± 11.89	62.83 ± 14.27	63.33 ± 10.03	59.65 ± 9.20	61.45 ± 12.94	NS
GLU (mmol/L)	Test	4.42 ± 0.66	4.42 ± 0.65	4.66 ± 1.01	4.88 ± 1.20	4.846 ± 0.9418	
	Control	4.20 ± 0.33	4.45 ± 0.66	3.76 ± 1.15	4.92 ± 0.456	4.624 ± 0.2368	NS
CK (mmol/L)	Test	107.6 ± 43.18	100.6 ± 42.68	98.13 ± 37.32	112.8 ± 35.4	111 ± 45.22	
	Control	90.2 ± 25.33	93.3 ± 28.72	98 ± 29.94	138.3 ± 135.9	108.3 ± 67.29	NS

*NS, Non-significant*.

### Efficacy

In the efficacy section, two time-points were regarded pre-treatment and post-treatment group. The pre-treatment group was the PBMCs from baseline or first infusion time-point, because at the time of blood sample collection, the participant was not exposed to therapy. The post-treatment group was the PBMCs of first follow-up time-point as this was the earliest time-point after the infusion of the cell product, whose result was able to show the acute effect of the therapy.

#### Effects on Immune Checkpoint Molecules

In this panel, the expression of three markers were tested by flowcytometry, which were PD-1, TIM-3 and CTLA-4. Changes of TIM-3 and CTLA-4 were not statistically significant (TIM-3 *p* value = 0.9780, CTLA-4 *p* value = 0.4577), PD-1 expression result showed a statistic significant downregulation, *p* value = 0.0448.

#### Effects on Differentiation of the Cells

CD45RA, CD45RO, CCR7, and CD27 were used to sort different subsets of the CD8+ cells, which were naïve cells, stem memory cells (TSCM), central memory cells (Tcm) and effector memory cells (Tem). The phenotypes for cell sorting are listed in Table 4.

**Table 4:** Expression of markers that differentiate each subset of memory cells.

**Naïve**	**TSCM**	**Tcm**	**Tem**
CD45RA+	CD45RA+	CD45RA−	CD45RA−
CD45RO−	CD45RO+	CD45RO+	CD45RO+
CCR7+	CCR7+	CCR7+	CCR7−
CD27+	CD27+	CD27+	CD27−

With a tendency of decrease on TSCM, Tcm and Tem, neither of these subset of memory cells achieved a statistic significance in the comparison of both groups (*p* value = 0.3484, 0.1064, and 0.1571, respectively), which was in line with Naïve cells (*p* value = 0.4954).

#### Effects on the Activation/Apoptosis of the Cells

In this panel, CD38, CD57, and CD95 were tested to measure the level of cell activation and apoptosis levels. The result showed no difference of CD38 and CD57 expression between both groups, indicating the activation of the overall CD8+ cells remained unchanged. However, the expression of CD95 was downregulated in the post-treatment group (*p* = 0.0258).

**Figure 3:**
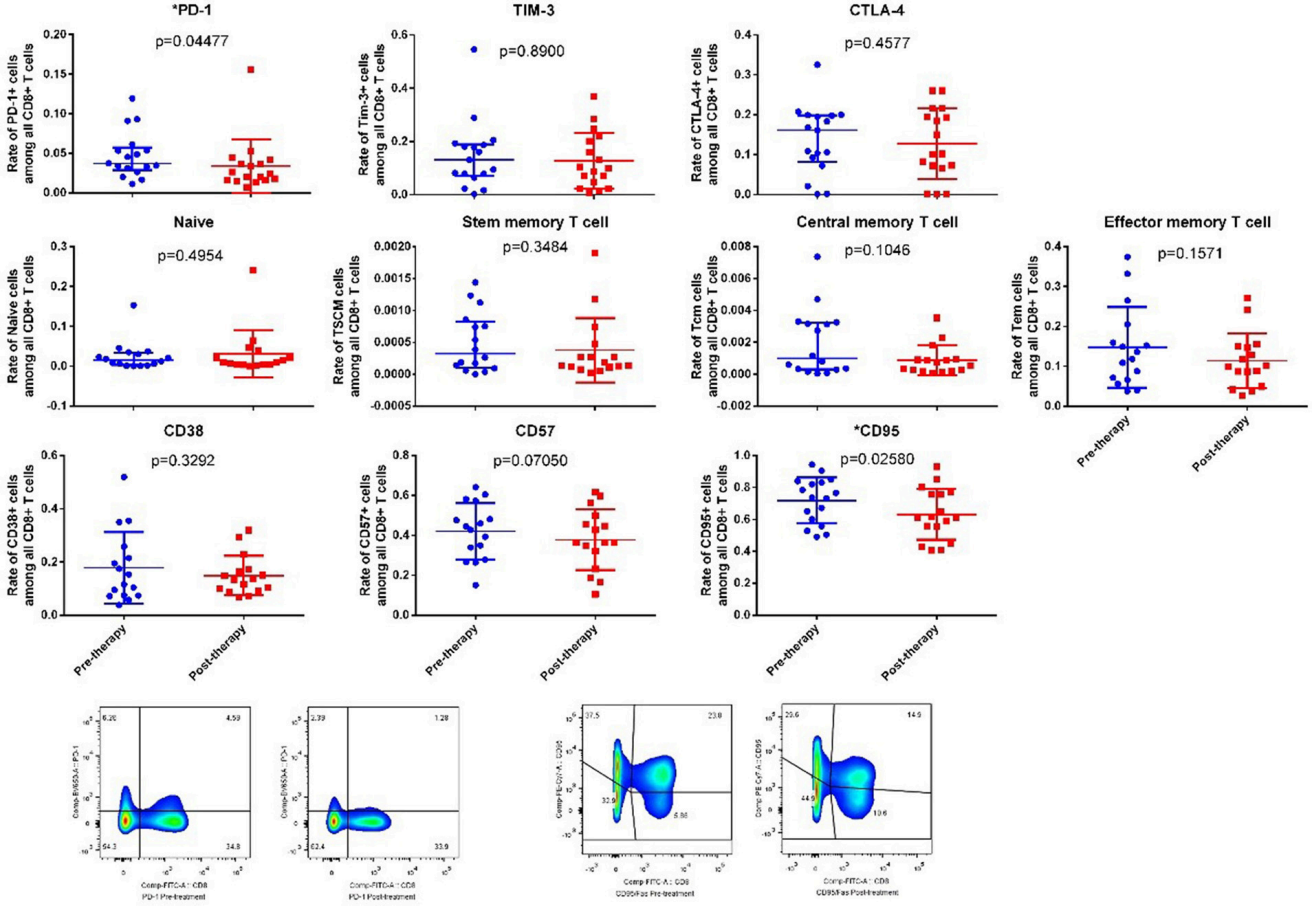
The changes of all the marker's expressions of laboratory evaluations of efficacy. Legend: Each dot represents an individual data, the horizontal line on each set of data represents the mean ± standard deviation (normal distribution data), or median, 25% quartile and 75% quartile (skewed distribution data).

Data of the efficacy section is showed in Table 5 and Figure 3.

**Table 5:** The changes of all the marker's expressions of laboratory evaluations of efficacy.

**Marker/subset**	**Pre-treatment group (%)**	**Post-treatment group (%)**	***p-*value**
PD-1	3.73 [2.89, 5.73]	2.39 [1.65, 3.94]	0.0448
TIM-3	13.06 [7.14, 18.95]	9.93 [3.50, 21.09]	NS
CTLA-4	16.11 [8.21, 19.82]	10.16 [6.93, 20.51]	NS
Naïve	1.55 [0.28, 3.39]	1.03 [0.41, 3.5]	NS
Tscm	0.03 [0.01, 0.08]	0.02 [0.01, 0.04]	NS
Tcm	0.10 [0.03, 0.3213]	0.061 [0.02, 0.09]	NS
Tem	14.75 ± 10.1	11.43 ± 6.86	NS
CD38	17.82 ± 13.39	14.88 ±7.40	NS
CD57	42.07 ± 14	37.85 ± 15.24	NS
CD95	71.82 ± 14.31	63.16 ± 15.96	0.0258

*NS, Non-significant*.

## Discussion

The investigation immunotherapy to eradicate HIV infection has never stopped. There have been several hypotheses of the strategies to do so. With the case of the “Berlin patient,” transplantation of bone marrow or stem cells that carries the HIV-resistant gene mutation seems to be a way to cure this infection. However, the risk of bone marrow transplantation along with very low natural occurrence of the favorable gene mutation are obstacles for replicating this case. Being another strategy, dendritic cell vaccine therapy which focuses on enhancing antigen presenting could be promising, however, existing immune exhaustion can severely compromise the function of the final effector immune cells, resulting in wasting antigen presentation enhancement. In that matter, T cells as one kind of the final effector has been investigated as a major tool for new therapies (9).

There are several key factors that support T cell therapies. Firstly, antigen specific T cells work as the central part of cellular adaptive immunity. Secondly, lymphocytes have the homing function which drives these cells to infiltrate to certain lymph tissues such as lymph node and lymphoid tissues (10), two compartments that are recognized HIV reservoir (11). Finally, the CTLs also have the ability to migrate to central nerve system which is also an important reservoir of the virus (12). Under these circumstances, cellular therapy based on CTLs has its great potential in curing HIV infection.

However, immune pressure from CTLs is an important factor that drives the virus to mutate (13), thus all the epitopes vulnerable to immune pressure cannot serve as targets for cellular therapy. But previous research has discovered the existence of highly conserved antigen epitopes of HIV (14). These epitopes are very resistant to mutation, despite long-term exposure to selective pressure from the immune system or irregular cART, which is likely to be one mechanism of the elite control status (15). The mechanism underlying this phenomenon is not clearly explained, one theory is that these epitopes belong to structural or key functional part of viral protein, the mutation of these epitopes would severely compromise the replication of the virus (16). In that matter, it is important to identify these conserved epitopes and to use them in future cellular therapies. Moreover, to maintain a long-term immune control of HIV, immune memory against these epitopes has to be formed to achieve a rapid reaction upon re-challenge of HIV antigens (17). However, there is a possibility that all though the course of the disease, the immune system of certain patients has not recognized any conserved epitope, let alone forming an immune memory. Even if immune memory against conserved epitopes has been formed, there are still several factors that could compromise the overall antiviral function.

Long term immune stimulation by circulating antigen can lead to several adverse immune changes including activation induced cell death (AICD) and upregulation of immune check point molecules. AICD is a mechanism against over-activation of immune system in the acute phase (18). Regulatory T cells (Treg) are the major effector in this mechanism. Treg binds the Fas-Ligand expressed on their surface to CD95/Fas molecules of the activated immune cells to induce apoptosis to prevent excessive activation (18). The down-regulatory immune check point molecules do the same job in acute phase of cell activation. However, previous observations have found that the expression of these molecules remained high after the acute phase and discovered the correlation between them and the exhaustion of T cells (19–21), highlighted the immune check point molecules in enhancing immune activity against infection or cancer.

The recognition of antigen epitopes is restricted by major histocompatibility complex (MHC) molecule which present these epitopes to effector immune cells. Human MHC haplotypes, also called human leukocyte antigen (HLA), distribute unevenly among or even within different ethnic groups (22, 23). In Han Chinese ethnic group, HLA-A02 is the most prevalently possessed HLA-A alleles which is up to half of the population (data based on *New allele frequency database*, www.allelefrequencies.net). Cellular therapy based on HLA-A02 restricted antigen epitopes thus can have a more prevalent usage in Chinese population. In this study, we developed a HLA-A02 restricted HIV antigen specific effector CTLs cellular therapy and tested its safety and efficacy.

In the safety section, patients only complained some minor side effects during the trial. Symptoms were recovered shortly after occurrence without medical intervention. Compared to some highly genetically modified cell therapies which have led to mortality (24), this result indicated that this therapy was safe.

For the efficacy aspect, no change in the immune memory was found based on sorting of subsets of CD8+ T cells. For immune check-point and maturation/activation, however, both PD-1 and CD95 expression had been downregulated post-treatment. It is reported in previous studies that the activation of CD95/Fas pathway induced apoptosis can be enhanced by PD-1, when PD-1 molecule migrated or expressed near the CD95 (25), thus up-regulation of both these molecules might have a stronger impact on immune suppression or exhaustion status. Unfortunately, the statistical significance of the downregulation of PD-1 in this trial was not very strong (*p* value = 0.0448), further investigation is needed to confer the impact on PD-1 expression of this trial.

There are also several limitations of this trial. Firstly, both the amount of participant and antigen specific CD8+ cells in each product were generally small. These two limitations hindered the therapy to give a maximum effect. Secondly, we did not anticipate the downregulation of both and only PD-1 and CD95, thus the design of efficacy evaluation flowcytometry did not include these two biomarkers within the same panel, which disabled us to investigate the percentage and number of cells that had downregulations of both markers. Finally, we only compared the changes of biomarkers of the test group, since all these patients were stable with cART. Nevertheless, this trial did show the safety of this autologous HIV-antigen specific effector CTLs cellular therapy and an impact on two cell markers which are correlated to immune suppression. The data of this trial might be able to provide useful reference to future cell therapy trials.

## Data Availability

The datasets generated and analyzed for this study can be found in the Chinese Clinical Trial Registry (ChiCTR-ICR-15005775), www.chictr.org.cn.

## Author Contributions

SL wrote the main body of the manuscript. SL and JS equally contributed to this work as first authors. YZ was the PI of this work. HW was in charge of the clinical department, provided the patient cohort, and supervised the clinical part of this trial. TD provided necessary scientific techniques and synthesis of key reagents. ZL, LQ, GL, and KL have contributed equally to the recruitment of patients, miscellaneous support to the work.

### Conflict of Interest Statement

The authors declare that the research was conducted in the absence of any commercial or financial relationships that could be construed as a potential conflict of interest.

## Supplementary Material

Supplementary Table 1 List of antibodies used in each panel.

Supplementary Table 2 Details of the cell products.

Supplementary Figure 1 Cut-off gating strategy for florescence minus one (FMO).

Supplementary Figure 2 Gating strategy for subset of CD8+ T cells.
