# Supplementary material for: T Cell Therapy Targeted on HLA-A02 Restricted HIV Antigen Epitopes: An Open Label Cellular Therapy Trial Using CD8+ T Cell
Source: Front Immunol. 2019 Mar 18;10:437. doi: 10.3389/fimmu.2019.00437 (PMC6435000; doi:10.3389/fimmu.2019.00437)
Supplement: Supplementary Table 1 — List of antibodies used in each panel. [file Table_1.docx]

Supplementary Table 1. List of antibodies used in each panel.

| Immune check-point panel | | | | Differentiation panel | | | | Activation/apoptosis panel | | | |
| --- | --- | --- | --- | --- | --- | --- | --- | --- | --- | --- | --- |
| Ab | Color | Clone | Manufacturer | Ab | Color | Clone | Manufacturer | Ab | Color | Clone | Manufacturer |
| CD3 | Alexa Fluor 700 | OKT3 | BioLegend, USA | CD3 | Alexa Fluor 700 | OKT3 | BioLegend, USA | CD3 | Alexa Fluor 700 | OKT3 | BioLegend, USA |
| CD4 | APC | RPA-T4 | BD, USA | CD4 | APC | RPA-T4 | BD, USA | CD4 | APC | RPA-T4 | BD, USA |
| CD8 | FITC | RPA-T8 | BD, USA | CD8 | FITC | RPA-T8 | BD, USA | CD8 | FITC | RPA-T8 | BD, USA |
| PD-1 | BV650 | 2D7 | BD, USA | CD45RA | BV421 | 5H9 | BD, USA | CD38 | BV605 | HB7 | BD, USA |
| TIM-3 | BV421 | 7D3 | BD, USA | CD45RO | PE-CF594 | UCHL1 | BD, USA | CD57 | BV421 | NK-1 | BD, USA |
| CTLA-4 | PE-Cy7 | L3D10 | BioLegend, USA | CCR7 | PE-Cy7 | 3D12 | BD, USA | CD95 | PE-Cy7 | BX2 | BioLegend, USA |
|  |  |  |  | CD27 | PE-Cy7 | O323 | BioLegend, USA | |  |  |  |
