# Supplementary material for: T Cell Therapy Targeted on HLA-A02 Restricted HIV Antigen Epitopes: An Open Label Cellular Therapy Trial Using CD8+ T Cell
Source: Front Immunol. 2019 Mar 18;10:437. doi: 10.3389/fimmu.2019.00437 (PMC6435000; doi:10.3389/fimmu.2019.00437)
Supplement: Supplementary Table 2 — Details of the cell products. [file Table_2.docx]

Supplementary Table 2. Details of the cell products.

| Pt.ID and Serial No. of product | | Days of  culture | Total cell  (x107) | %live cell | %CD3+ | %CD3+CD8+IFN-γ+ | Count of  CD3+CD8+IFN-γ+  cell (x105) | P24 and endotoxin level |
| --- | --- | --- | --- | --- | --- | --- | --- | --- |
| HBX | HIVA2-00101-20150520 | 13 | 3.52 | 94.6 | 76.31 | 0.4 | 1.01 | UCO |
|  | HIVA2-00102-20150602 | 13 | 7.22 | 91.7 | 81.56 | 0.5 | 2.70 | UCO |
| MJ | HIVA2-00201-20150707 | 20 | 4 | 75 | 88.35 | 0 | 0 | UCO |
|  | HIVA2-00202-20150727 | 14 | 4.95 | 90 | 68.57 | 0.6 | 1.83 | UCO |
| WXF | HIVA2-00301-20150714 | 14 | 8.95 | 96.7 | 87.2 | 3.2 | 24.15 | UCO |
|  | HIVA2-00302-20150728 | 13 | 9.74 | 94.9 | 86.5 | 3.5 | 27.98 | UCO |
| WHC | HIVA2-00501-20150717 | 13 | 5 | 90.9 | 86.09 | NA | NA | UCO |
|  | HIVA2-00502-20150730 | 16 | 31.5 | 96.92 | 72.35 | NA | NA | UCO |
| ZZY | HIVA2-00601-20150724 | 14 | 10.5 | 95.5 | 82.64 | 0.9 | 7.46 | UCO |
|  | HIVA2-00602-20150807 | 13 | 7.5 | 93.75 | 81.27 | 1.7 | 9.71 | UCO |
| WYP | HIVA2-00701-20150724 | 14 | 11.1 | 95.7 | 59.18 | 2.7 | 16.97 | UCO |
|  | HIVA2-00702-20150807 | 13 | 10.5 | 95.7 | 59.06 | 3.1 | 18.40 | UCO |
| ZZQ | HIVA2-00803-20150910 | 14 | 15.4 | 96.5 | 68.1 | 0.2 | 2.02 | UCO |
|  | HIVA2-00804-20150924 | 16 | 22.5 | 90 | 90.2 | 0.3 | 5.48 | UCO |
| MQQ | HIVA2-00901-20150730 | 14 | 13.5 | 96.43 | 74.59 | 10.9 | 105.84 | UCO |
|  | HIVA2-00902-20150813 | 13 | 8 | 97.56 | 79.62 | 9.1 | 56.55 | UCO |
| YGJ | HIVA2-01301-20150805 | 14 | 6.79 | 91.2 | 85.2 | NA | NA | UCO |
|  | HIVA2-01302-20150819 | 14 | 8.01 | 90.9 | 88.1 | NA | NA | UCO |
| CB | HIVA2-01402-20150805 | 14 | 3.95 | 89.4 | 76.4 | NA | NA | UCO |
|  | HIVA2-01403-20150819 | 14 | 4.36 | 90.1 | 79.2 | NA | NA | UCO |
| GJL | HIVA2-01101-20150812 | 14 | 14 | 93.3 | 78.3 | 1.2 | 12.27 | UCO |
|  | HIVA2-01103-20150910 | 14 | 13.75 | 89.3 | 66.8 | 0.1 | 0.82 | UCO |
| PYH | HIVA2-01501-20150907 | 16 | 46.75 | 95.51 | 74.18 | 0.2 | 6.62 | UCO |
|  | HIVA2-01502-20150923 | 16 | 15.5 | 91.2 | 59 | 0.3 | 2.50 | UCO |
| XWJ | HIVA2-01601-20150907 | 16 | 37.4 | 95.77 | 46.41 | 2.4 | 39.89 | UCO |
|  | HIVA2-01602-20150923 | 16 | 19 | 95 | 64.5 | 0.5 | 5.82 | UCO |
| HJJ | HIVA2-01701-20150923 | 17 | 17.5 | 89.7 | 92.1 | 0.1 | 1.45 | UCO |
|  | HIVA2-01702-20151010 | 16 | 7 | 87.5 | 65.4 | 0.1 | 0.40 | UCO |
| LTS | HIVA2-01801-20150925 | 15 | 8 | 84.2 | 92.4 | 0.1 | 0.62 | UCO |
|  | HIVA2-01802-20151010 | 13 | 4 | 93.2 | 79 | 0.1 | 0.29 | UCO |
| DW | HIVA2-01901-20151013 | 15 | 8 | 94.1 | 83.5 | 0 | 0 | UCO |
|  | HIVA2-01902-20151028 | 14 | 20 | 97.5 | 67.5 | 1 | 13.16 | UCO |
| ZL | HIVA2-02001-20151013 | 15 | 10 | 90.9 | 83.9 | 0.1 | 0.76 | UCO |
|  | HIVA2-02002-20151028 | 14 | 13 | 89.65 | 69.9 | 0.2 | 1.63 | UCO |

UCO: Under cut off value
