# Supplementary figures and images for: T Cell Therapy Targeted on HLA-A02 Restricted HIV Antigen Epitopes: An Open Label Cellular Therapy Trial Using CD8+ T Cell
Source: Front Immunol. 2019 Mar 18;10:437. doi: 10.3389/fimmu.2019.00437 (PMC6435000; doi:10.3389/fimmu.2019.00437)

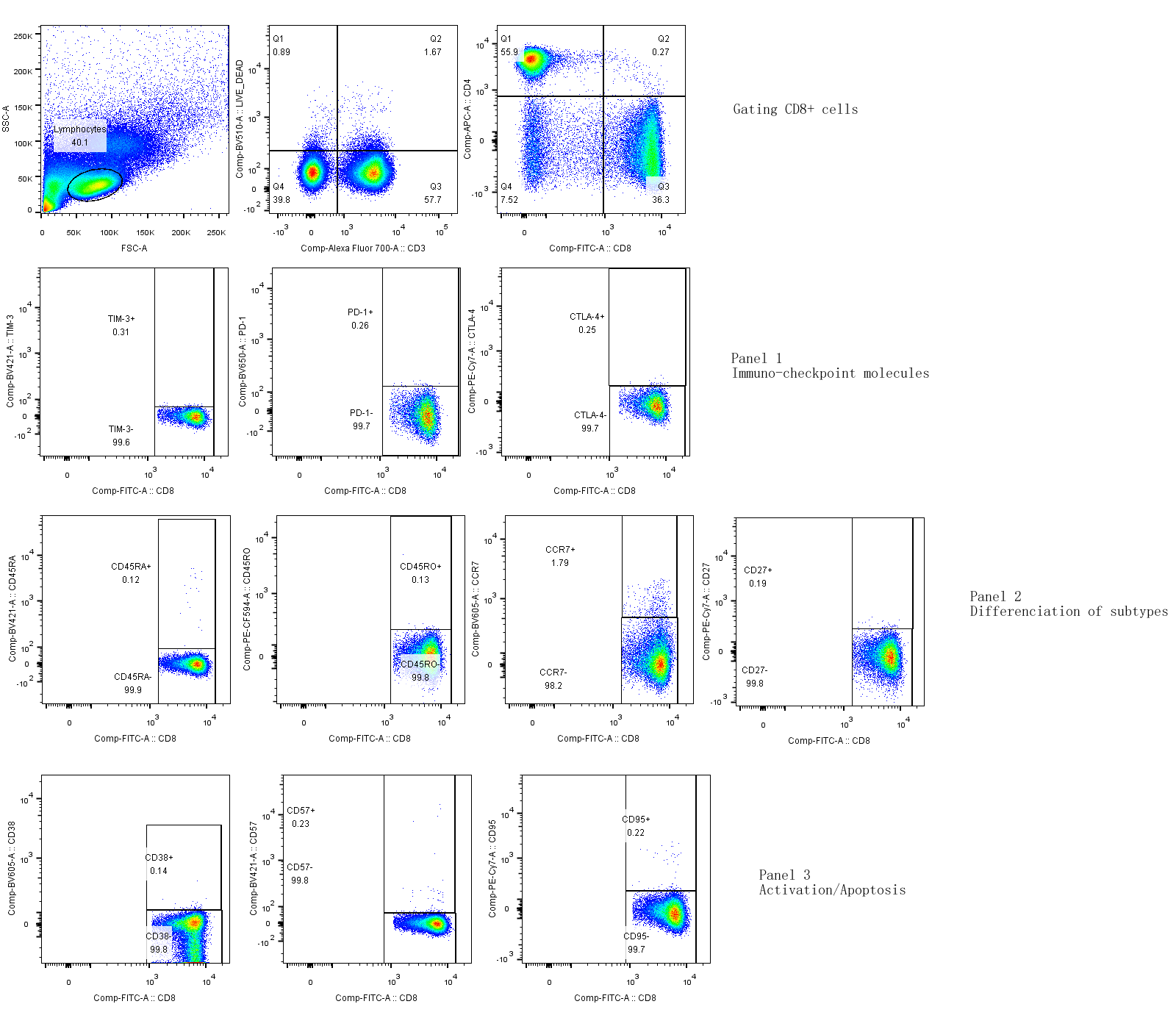

Supplement: Supplementary Figure 1 — Cut-off gating strategy for florescence minus one (FMO). [file Image_1.png]

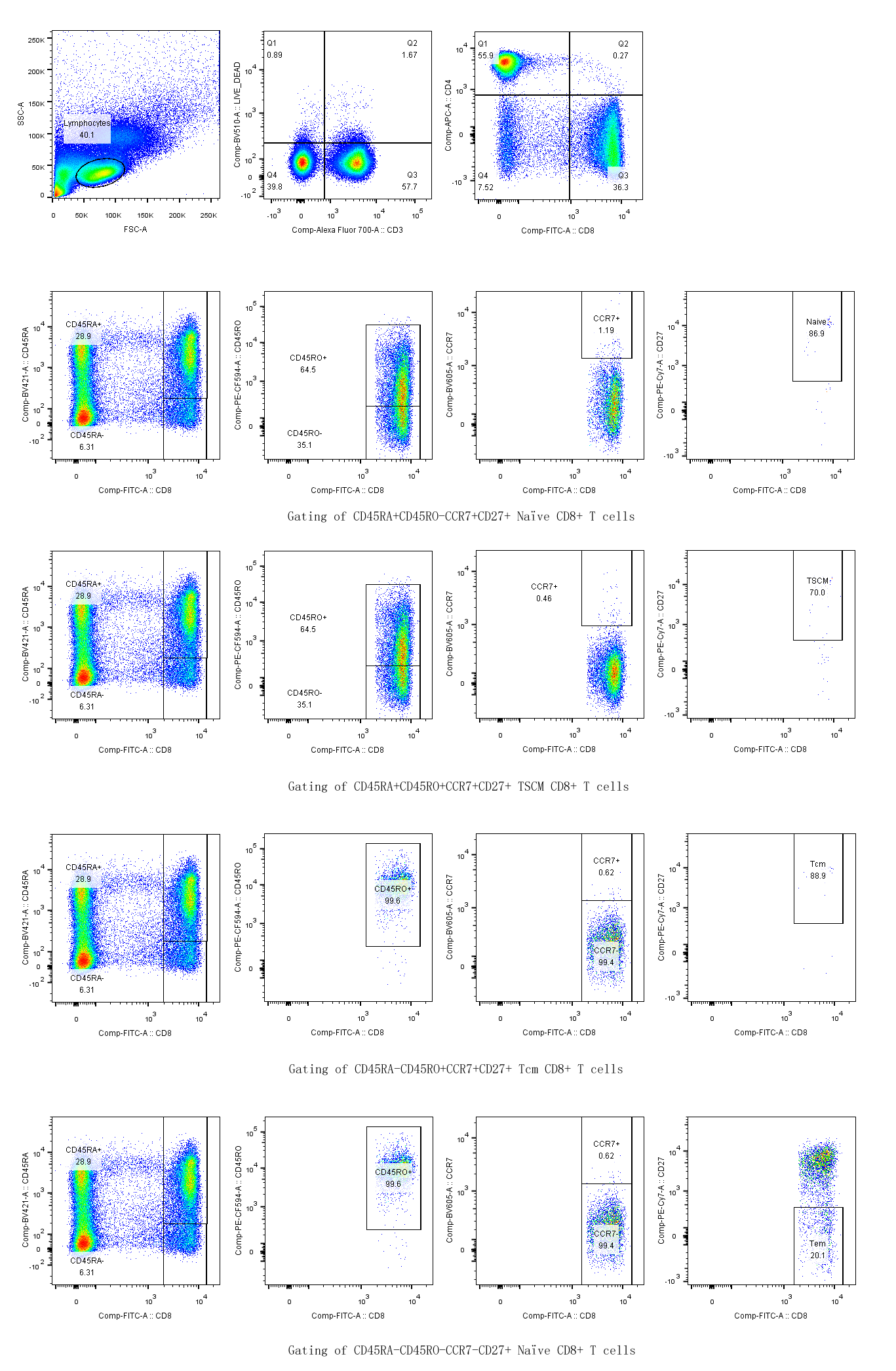

Supplement: Supplementary Figure 2 — Gating strategy for subset of CD8+ T cells. [file Image_2.png]
